# Can acetate via FFA receptors contribute to the diabetogenic effect of statins?

**DOI:** 10.1007/s00210-023-02647-7

**Published:** 2023-08-29

**Authors:** Finn Olav Levy, Jan-Bjørn Osnes

**Affiliations:** Department of Pharmacology, Division of Laboratory Medicine, Institute of Clinical Medicine, University of Oslo and Oslo University Hospital, Sognsvannsveien 20, P.O. Box 1057 Blindern, N-0316 Oslo, Norway

**Keywords:** Lipid-lowering agents, HMG-CoA reductase inhibitors, Side effect, Diabetes, Cholesterol, Bempedoic acid

## Abstract

Despite the proven effects of statins in preventing cardiovascular disease, their diabetogenic effect has caused concern. The mechanism of this diabetogenic effect is unknown. We suggest a novel mechanism that may contribute to the diabetogenic effect of statins, through an effect of statins that has apparently escaped previous consideration. Briefly, by inhibiting HMG-CoA reductase, statins may cause accumulation of acetate, which through FFA2 and FFA3 stimulation may inhibit insulin secretion.

Although the benefits of statins in preventing cardiovascular events by far outweigh their diabetogenic effect, the latter has caused considerable concern due to a 10–25% increased risk of new-onset diabetes (Thakker et al. 2016; Carmena and Betteridge 2019; Satter 2023). The extensive network meta-analysis by Thakker et al. (2016) covers and tabulates relevant studies on the diabetic risk of statins. The risk is estimated to be 10–20 times that of developing myopathy on statin treatment (Satter 2023). The diabetogenic effect is considered a class effect of statins that is not shared by other LDL cholesterol–reducing agents (Thakker et al. 2016; Satter 2023). The largest risk increase is in patients with components of metabolic syndrome on intensive statin treatment. The mechanisms of this diabetogenic effect of statins are far from clear but seem to involve reduction of both insulin secretion and insulin sensitivity (Carmena and Betteridge 2019). Weight gain during the statin treatment period seems to account for about half of the diabetic risk (Satter 2023; Yang and Schooling 2023). Nevertheless, other mechanisms for the diabetogenic effect of statins have been sought (Satter 2023; Yang and Schooling 2023). Although a potential epigenetic mechanism was put forward, the authors state that “other biological pathways should not be discarded” (Ochoa-Rosales et al. 2020). As statins are inhibitors of the hydroxymethylglutaryl-coenzyme A (HMG-CoA) reductase, possible diabetogenic mechanisms have primarily been sought downstream of this enzyme (Carmena and Betteridge 2019). However, HMG-CoA reductase inhibition may also cause upstream changes, such as accumulation of both HMG-CoA and the precursor acetyl-CoA (Allen and Marnotte 2017; Baul et al. 2020). Statin-induced elevation of ketones in patients with type 2 diabetes has been observed and tentatively explained by an increase in acetyl-CoA (Baul et al. 2020). Although Sliz et al. (2018) did not find an increase of ketones during treatment with pravastatin, an increase of acetyl-CoA is not excluded by their findings. The recently approved LDL cholesterol–reducing agent bempedoic acid inhibits ATP-citrate lyase and thus the synthesis of acetyl-CoA (Burke et al. 2019). Bempedoic acid does not increase the diabetes risk—if anything, it decreases the risk (Satter 2023; Rusica et al. 2022). This may provide some indirect support for a diabetogenic role of increase in acetyl-CoA. Acetyl-CoA is also a source for acetate production (Tang et al. 2015). Acetate is an endogenous agonist of the G-protein-coupled receptors for short-chain fatty acids, FFA2 and FFA3, present on pancreatic beta cells (Tang et al. 2015; Liu et al. 2020). These receptors are available to acetate and are involved in the regulation of insulin secretion. Both receptors are coupled to Gα_i_, inhibiting adenylyl cyclase and thus reducing cAMP synthesis. This will lower insulin secretion (Rusica et al. 2022). Although FFA2 is also coupled to the Gα_q_ pathway, which may increase insulin secretion (Liu et al. 2020), the net effect of acetate stimulation of FFA2 and FFA3 is considered to be a decreased insulin secretion (Rusica et al. 2022), although contradictory results exist (Liu et al. 2020). By combining these findings, an additional and novel mechanism for the reduction of insulin secretion by statins came to our mind, a mechanism that surprisingly had not been mentioned in any publications (Fig. 1): Statins may cause accumulation of acetyl-CoA leading to elevation of acetate at least locally in and around the pancreatic beta cells. Increased acetate could enhance stimulation of FFA2 and FFA3 on the beta cells, thus reducing insulin secretion through decreased cAMP. We suggest two approaches for clinical studies possibly clarifying this mechanism for the diabetic risk of statins (Table 1):

1. Combining statins with FFA2 and FFA3 receptor antagonists, which are under development (Liu et al. 2020). A counteraction of the diabetic risk by these agents would support our hypothesis.
2. Combining statins with bempedoic acid which should counteract an accumulation of acetyl-CoA caused by statins (Fig. 2). Biomarkers for diabetes and prediabetes should be measured. The only study found so far on this combination did not include such measurements (Rusica et al. 2022).

**Fig. 1:**
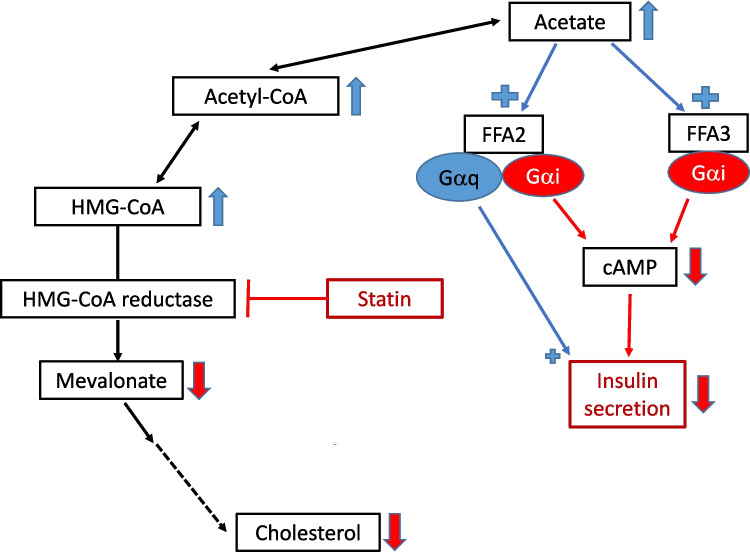
A suggested mechanism of action contributing to the diabetogenic effect of statins. By inhibiting HMG-CoA reductase, statins increase acetyl-CoA and acetate. Blue arrows and red arrows indicate increase and decrease, respectively. Blue plus signs indicate stimulation

**Table 1:** Suggested clinical studies possibly clarifying an involvement of acetyl-CoA and acetate as a mechanism of action contributing to the diabetogenic action of statins

Study approach	Rationale	Interpretation
Combining statins with FFA2 and FFA3 receptor antagonists, which are under development (Liu et al. 2020)	Blockade of these receptors would inhibit an effect of receptor stimulation by acetate	A reduction of the statin-induced diabetic risk by these receptor antagonists would support the hypothesis of an involvement of acetate
Combining statins with the new cholesterol lowering agent bempedoic acid	Bempedoic acid acts by inhibiting ATP-citrate lyase-catalyzed conversion of citrate to acetyl-CoA (Burke et al. 2019). Thus, it should counteract the increase in acetyl-CoA caused by statins (Fig. 2)	A reduction of the statin-induced diabetic risk by bempedoic acid would support the hypothesis of an involvement of acetyl-CoA/acetate

**Fig. 2:**
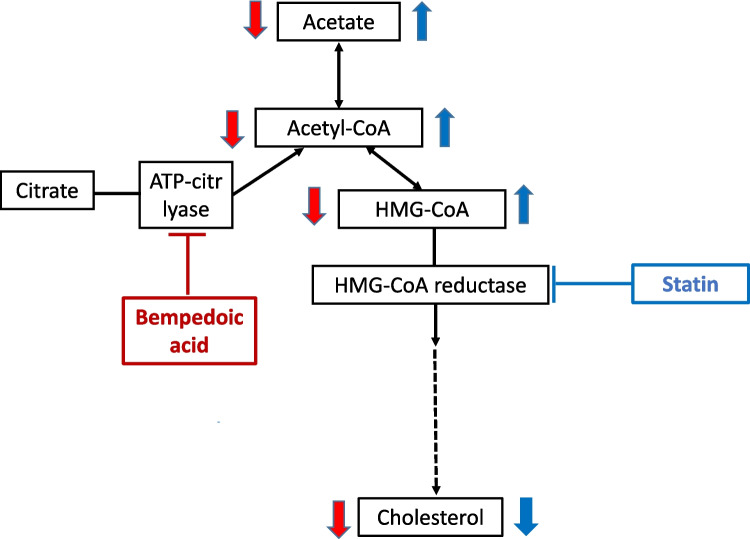
Interaction between the two cholesterol lowering agents, statins and bempedoic acid. By inhibiting ATP-citrate lyase, bempedoic acid acts upstream of statins and thus decreases acetyl-CoA and acetate. We assume this to counteract the increasing effect of statins. Blue symbols: effects of statins. Red symbols: effects of bempedoic acid

## Data Availability

Not applicable
